# A practical nomogram based on serum interleukin-6 for the prognosis of liver failure

**DOI:** 10.3389/fmed.2022.1035699

**Published:** 2022-11-16

**Authors:** Nanxi Xiao, Linxiang Liu, Yue Zhang, Yuan Nie, Xuan Zhu

**Affiliations:** Department of Gastroenterology, The First Affiliated Hospital of Nanchang University, Nanchang, China

**Keywords:** liver failure, inflammatory factors, diagnosis, prognosis, systemic inflammation

## Abstract

**Background:**

Liver failure (LF) is a serious liver function damage caused by various factors, mainly jaundice, hepatic encephalopathy, coagulation disorders and multiple organ failure, with the clinical characteristic of high short-term mortality. LF is often accompanied by excessive activation of inflammatory factors, and an excessive systemic inflammatory response (i.e., inflammatory storm) is considered to be the trigger of LF. However, a specific prognostic model including inflammatory factors for patients with LF has not been well established.

**Aim:**

To establish and validate a nomogram for predicting 28-day, 90-day, and 180-day mortality in patients with LF.

**Methods:**

A total of 423 eligible LF patients were enrolled in this retrospective study. Independent predictors were identified using a multivariate logistic model and then integrated into a nomogram to predict 28-day, 90-day, and 180-day mortality. The concordance index, receiver operating characteristic curves, and calibration plots were used to evaluate the performance of the model.

**Results:**

Sex, age, total bilirubin, aspartate aminotransferase, international normalized ratio, Child–Pugh score, and serum interleukin-6 were independent risk factors for death at 28, 90, and 180 days in LF patients. The nomogram showed good calibration and discrimination with an area under the receiver operating characteristic curve (AUC) of 0.927. The calibration curve fit as well, indicating that the nomogram had good clinical application value.

**Conclusion:**

This nomogram model for predicting the 28-day, 90-day, and 180-day mortality of LF patients could help optimize treatment strategies and improve prognosis.

## Introduction

Liver failure (LF) can be caused by many factors [direct toxic necrosis or apoptosis and immune damage (1)] and mainly manifests as jaundice, hepatic encephalopathy, coagulation dysfunction and multiple organ failure, with high short-term mortality. Acute liver failure (ALF) can be divided into three types, including ALF without liver disease, acute-on-chronic liver failure (ACLF) on the basis of chronic liver disease or cirrhosis, and acute decompensation of cirrhosis. The ALF classification applies to a unique and uncommon severe hepatocellular damage syndrome that causes clotting and altered intellectual ability in the absence of chronic liver disease. ACLF is defined by the Asian-Pacific Association for the Study of the Liver (APASL) as acute liver injury presenting with jaundice and coagulation disorders, ascites and/or hepatic encephalopathy within 4 weeks in patients with previously diagnosed or undiagnosed chronic liver disease. Decompensated cirrhosis is defined as the presence or history of ascites, hemorrhage, hepatic encephalopathy, or jaundice on top of cirrhosis (2). The above three types of LF are often accompanied by excessive activation of inflammatory factors, and an excessive systemic inflammatory response (also known as an inflammatory storm) is considered to be the acute triggering factor of LF. Systemic inflammation is mainly manifested by a significant increase in plasma proinflammatory factors, white blood cell counts and C-reactive protein (CRP), but the underlying mechanism is not fully understood. Organ failure and inflammatory storms play an important role in the pathogenesis of LF. It has been found that a variety of immune cells and proinflammatory and anti-inflammatory cytokines are involved in the occurrence and development of LF. Therefore, studying the changes in cytokines in the peripheral blood of patients with LF is helpful to understand the immune status of patients with LF and further predict the prognosis of patients with LF.

Some studies have proposed a hypothesis based on systemic activation of the coagulation system in response to the dysregulation of inflammatory markers (3–5). Interleukin 6 (IL-6) is a pleiotropic cytokine produced in response to infection and tissue damage. IL-6 stimulates Toll-like receptor 4 synthesis and secretion by lipopolysaccharide, IL-1β, or tumor necrosis factor-α and is one of the major stimulators of the release of liver acute phase proteins such as CRP, complement C3, fibrinogen, thromboplastin and serum amyloid A. IL-6 has an important immunomodulatory function. When infection or inflammation occurs in the body, its level will increase rapidly in a short period of time, which can be used as an immunological indicator for the early diagnosis of infection (6). Several studies in recent years have shown that high levels of IL-6 are associated with increased mortality of hepatitis B-associated acute-on-chronic liver failure [HBV-ACLF (7)], renal insufficiency in decompensated cirrhosis (8), hepatic encephalopathy complicated by cirrhosis (9), and increased mortality of end-stage liver disease (10). However, no study has explored the role of IL-6 in the prognosis of patients with LF.

The existing Child–Turcotte–Pugh (CTP) and Model for End-Stage Liver Disease (MELD) are both recognized liver disease assessment models, but both ignore immune dysfunction, which is closely related to the prognosis of LF patients. Although the neutrophil-to-lymphocyte ratio (NLR) score takes into account immune cell counts, it does not incorporate inflammatory cytokines. This study aimed to establish a new prediction model based on existing scores and cytokines for evaluating the prognosis of patients with LF from the perspective of inflammatory storms, guiding clinical diagnosis and treatment and providing accurate screening and rapid and active clinical intervention to improve the survival rate of patients and prevent wasting clinical resources.

## Materials and methods

### Patient selection

**FIGURE 1:**
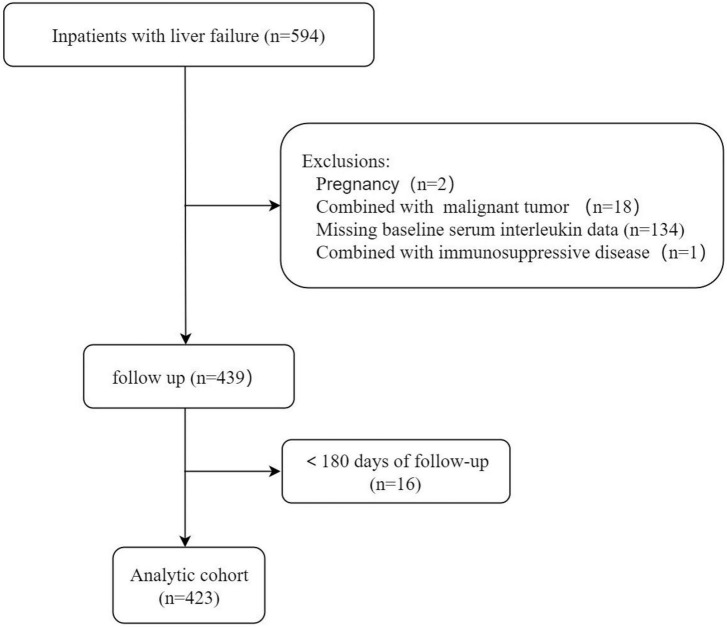
Patient selection flowchart.

This observational retrospective study was conducted on patients with LF admitted to the First Affiliated Hospital of Nanchang University from May 2019 to March 2021. LF was defined as (11–14) (1) extreme fatigue accompanied by severe gastrointestinal symptoms such as apparent anorexia, abdominal distension, nausea, and vomiting; (2) jaundice progressively worsening within 4 weeks, with serum total bilirubin (TBIL) ≥ 85.5 μmol/L or daily elevation ≥ 17.1 μmol/L; and (3) hemorrhagic tendency, prothrombin activity (PTA) ≤ 40%, or international normalized ratio (INR) ≥ 1.5, excluding extrahepatic causes. Cirrhosis was diagnosed by hepatic encephalopathy, variceal hemorrhage, imaging, hepatorenal syndrome or liver biopsy. The extent of ascites was diagnosed according to the International Ascites Association (15) and the American Association for the Study of Liver Diseases. The inclusion criteria were as follows: (1) patients aged over 18 years; (2) patients meeting the diagnostic criteria for LF (on account of clinical, laboratory, imaging examinations, transient elastography results or biopsy confirmations); and (3) patients hospitalized for at least 1 day. The exclusion criteria were as follows: (1) infection with human immunodeficiency virus; (2) primary liver carcinoma or other malignant tumors with or without metastasis; (3) concurrent pregnancy; (4) receiving immune-suppressive medication; (5) lost to follow-up or incomplete data; (6) liver transplantation; and (7) other serious chronic extrahepatic diseases, such as renal failure and heart failure. The study flow chart is illustrated in Figure 1.

All patients were given comprehensive medical treatment, including artificial liver support if necessary, hemostatic endoscopy or drug treatment, antiviral and nutritional support, albumin supplementation or fresh plasma, maintenance of water electrolyte and acid–base balance, maintenance of microecological stability, prevention and treatment of complications, etc. The prognosis of the enrolled patients with LF was followed up until September 30, 2021, to ensure that the last patient (who was enrolled on March 30, 2021) was also followed up for at least 6 months. The follow-up methods were as follows: (1) telephone follow-up and (2) clinical follow-up (including outpatient and inpatient visits).

### Data collection

The data included the demographic status, etiology of the liver disease, and clinical laboratory tests. Clinical laboratory tests were completed within 24 h of admission, including hemoglobin (Hb), platelet count (PLT), white blood cell count (WBC), neutrophil count, lymphocyte count, HBV test, IL-6, D-dimer, prothrombin time (PT), INR, albumin (ALB), serum TBIL, alanine aminotransferase (ALT), aspartate aminotransferase (AST), gamma-glutamyl transferase (GGT), blood electrolytes, blood lipids, blood urea nitrogen (BUN), serum creatinine (CRE), MELD score, Child–Pugh score, etc. The relevant scoring formula was calculated as follows: the NLR was calculated as neutrophil count/lymphocyte count (10^9^/L). The Institutional Review Board of the First Affiliated Hospital of Nanchang University (IIT [2021]009) approved the use of medical record data for this study.

## Statistical analysis

Categorical variables are expressed as frequencies and percentages, and quantitative variables are expressed as the mean ± standard deviation. The logarithm of IL-6 is expressed as LN (IL-6), so that it could be normalized and analyzed easily. The independent-sample test was used to compare two means, and the correlation analysis was based on Pearson correlation analysis (when both variables conformed to a normal distribution) or Spearman’s correlation analysis. Non-normally distributed variables are expressed as the median and interquartile range, and significance was determined using the Mann–Whitney *U* test. Univariate logistic regression analysis was performed to screen candidate indicators for risk factors from the prognosis-related assay indicators. The independent variables with *P* < 0.05 in univariate analysis were analyzed by multivariate logistic regression. The discrimination of the nomogram was measured by calculating the area under the receiver operating characteristic curve (AUROC) and the concordance index. Differences were considered significant at *P* < 0.05. SPSS 26.0 statistical software package and R version 4.1.2 were used for analysis.

## Results

### Baseline characteristics of patients with liver failure

As shown in Table 1, 423 patients with LF were admitted to the hospital between May 2019 and March 2021. The demographic and biochemical characteristics of the study population are outlined in Table 1. The mean (± standard deviation) age of the 423 patients was 48.5 (± 13.2) years. The majority of the patients were male (345/423). Among the patients included in the analysis, 39.7% had decompensated cirrhosis, and 54.1% did not have obvious evidence of ascites. The leading cause for hospitalization was jaundice (309/423), followed by ascites (72/423), infection (23/423), hepatic encephalopathy (12/423), gastrointestinal hemorrhage (6/423), and oliguria (1/423). A total of 22.9% of patients died within 28 days of admission, and 31% died within 180 days of admission.

**TABLE 1:** Baseline characteristics of patients with LF.

Variable	Patients with liver failure (*n* = 423)
Sex (male), *n* (%)	345 (81.5%)
Age, mean ± SD	48.5 ± 13.2
Decompensated cirrhosis, *n* (%)	168 (39.7%)
**Cause of liver failure, *n* (%)**	
Viral	348 (82.3%)
Alcoholic	6 (1.4%)
Combined alcoholic + viral	20 (4.7%)
Other	18 (4.2%)
Cryptogenic	31 (7.3%)
**Cause of hospitalization, *n* (%)**	
Ascites	72 (17.0%)
Gastrointestinal hemorrhage	6 (1.4%)
Hepatic encephalopathy	12 (2.8%)
Infection	23 (5.4%)
Jaundice	309 (73.0%)
Oliguria	1 (0.2%)
**Ascites degree**	
No ascites	229 (54.1%)
1 degree ascites	130 (30.7%)
2 degree ascites	50 (11.8%)
3 degree ascites	15 (3.5%)
Acute renal failure, *n* (%)	46 (10.9%)
**Therapy, *n* (%)**	
Vasopressor support	27 (6.4%)
Mechanical ventilation	2 (0.5%)
Liver replacement therapy	192 (45.4%)
Endoscope therapy	4 (0.9%)
**28-day outcome, *n* (%)**	
Survivors	326 (77.1%)
Non-survivors	97 (22.9%)
**90-day outcome, *n* (%)**	
Survivors	309 (73.0%)
Non-survivors	114 (27.0%)
**180-day outcome, *n* (%)**	
Survivors	292 (69.0%)
Non-survivors	131 (31.0%)

### Comparing the laboratory characteristics between the non-surviving and surviving groups

The clinical and laboratory characteristics of the patients are listed in Table 2. According to the 28-day results, the LF patients were divided into the non-surviving (*n* = 97) and surviving (*n* = 326) groups. Most non-survivors had higher laboratory parameters than survivors, as evidenced by AST, bilirubin, INR, PT, D-dimer, WBC, Child–Pugh score, MELD score, MELD-Na score, NLR, IL-6, and LN (IL-6) (*P* < 0.05). However, non-survivors had lower ALB, PLT, and serum Na levels than survivors (*P* < 0.05). ALT, alkaline phosphatase (ALP), creatinine, and GGT were not significantly different between the two groups (*P* < 0.05). The results at 3 and 6 months of follow-up were essentially the same as those at 28 days, except for creatinine, which was not statistically significant. There were significant differences in mortality at 28 days, 90 days, and 180 days after receiving artificial liver support. A greater proportion of surviving patients received artificial liver support therapy in Supplementary Table 1.

**TABLE 2:** Comparing the laboratory characteristics between non-surviving groups and surviving groups.

Parameter	28-day			90-day			6-month		
			
	Survivors	Non-survivors	*P*-value	Survivors	Non-survivors	*P*-value	Survivors	Non-survivors	*P*-value
ALT, IU/L	352 (93–856)	452 (132–1190)	0.133	352.0 (93–874)	434.5 (118.8–1173)	0.343	366.5 (93.5–948.5)	362 (103–1117)	0.910
AST, IU/L	210 (92–494.4)	348 (167.0–966.6)	**< 0.001**	215 (92–494.4)	293 (152–936.3)	**0.002**	216.9 (92–515.5)	274 (148.3–703)	**0.017**
Albumin, g/L	33.3 (29.4–36.7)	31.2 (28.7–33.3)	**< 0.001**	33.3 (29.4–36.6)	31.4 (28.9–33.6)	**< 0.001**	33.4 (29.5–36.8)	31.3 (28.7–33.7)	**< 0.001**
Bilirubin, μmol/L	192.5 (85.2–309.3)	330.1 (220.5–424.4)	**< 0.001**	187.6 (83.6–300.3)	328.1 (217.2–427.8)	**< 0.001**	189.9 (82.9–297.4)	309.3 (200.8–424.4)	**< 0.001**
ALP, IU/L	151.4 (122–194)	148 (121.0–194)	0.943	151.4 (120–194)	151 (122.3–194)	0.709	151.4 (119.5–192.5)	151.0 (122.3–203.5)	0.525
GGT, IU/L	120 (72–186)	978.0 (68–156.0)	0.115	124 (74–188)	95 (65–152)	**0.009**	124.5 (76–190)	91 (60.0–153.0)	**0.001**
Creatinine, μmol/L	63.5 (53.8–76)	68.3 (57.5–89.9)	**< 0.001**	63.5 (53.9–75.6)	66.8 (53.9–89.9)	0.051	63.6 (54.1–76.1)	66.1 (53.0–87.4)	0.168
INR	1.43 (1.21–1.76)	2.15 (1.79–2.98)	**< 0.001**	1.41 (1.2–1.73)	2.13 (1.75–2.97)	**< 0.001**	1.40 (1.18–1.70)	2.12 (1.67–2.89)	**< 0.001**
PT	16.2 (13.9–19.5)	23.9 (19.3–32.0)	**< 0.001**	16.1 (13.7–19.3)	23.65 (19.1–31.9)	**< 0.001**	15.9 (13.6–18.95)	23.5 (19.0–30.9)	**< 0.001**
D-dimer	0.95 (0.43–2.5)	3.48 (1.42–5.11)	**< 0.001**	0.94 (0.42–2.43)	3.13 (1.25–5.09)	**< 0.001**	0.92 (0.4–2.38)	2.93 (1.18–5.03)	**< 0.001**
Platelets, 10*9/L	121 (85–168)	89 (68–136)	**< 0.001**	124 (85–168)	93 (66–138)	**< 0.001**	126 (87–173)	93 (61–136)	**< 0.001**
WBC, 10*9/L	5.35 (4.11–7.21)	6.67 (4.81–9.07)	**< 0.001**	5.33 (4.1–7.1)	6.70 (4.85–9.37)	**< 0.001**	5.32 (4.07–7.07)	6.62 (4.77–9.07)	**< 0.001**
Na, mmol/L	138.6 (136.5–140.6)	136.9 (133.3–139.2)	**< 0.001**	138.6 (136.7–140.6)	136.9 (133.0–139.1)	**< 0.001**	138.7 (136.8–140.7)	137.1 (133.4–139.1)	**< 0.001**
Child–Pugh score	9 (7–11)	12 (11–12)	**< 0.001**	9 (7–10)	12 (11–12)	**< 0.001**	9 (7–10)	12 (10–12)	**< 0.001**
MELD score	16 (12–20)	24 (21–28)	**< 0.001**	15 (11–20)	24 (21–27)	**< 0.001**	15 (11–19)	23 (20–27)	**< 0.001**
MELD-Na score	16 (12–21)	26 (21–30)	**< 0.001**	16 (12–21)	25 (21–30)	**< 0.001**	16 (12–20)	24 (21–30)	**< 0.001**
NLR	2.69 (1.79–4.39)	5.43 (3.28–7.80)	**< 0.001**	2.64 (1.75–4.13)	5.28 (3.28–7.67)	**< 0.001**	2.59 (1.73–4.1)	4.93 (3.09–7.65)	**< 0.001**
IL-6	7.78 (3.98–16.48)	17.86 (8.54–30.09)	**< 0.001**	7.64 (3.98–15.53)	16.73 (8.29–32.96)	**< 0.001**	7.705 (3.97–15.625)	16.38 (7.44–32.81)	**< 0.001**
LN (IL-6)	2.05 (1.38–2.8)	2.88 (2.14–3.67)	**< 0.001**	2.03 (1.38–2.74)	2.82 (2.12–3.5)	**< 0.001**	2.05 (1.38–2.75)	2.80 (2.01–3.49)	**< 0.001**

Bold values represent the statistically significant.

### Univariate and multivariate analyses

**FIGURE 2:**
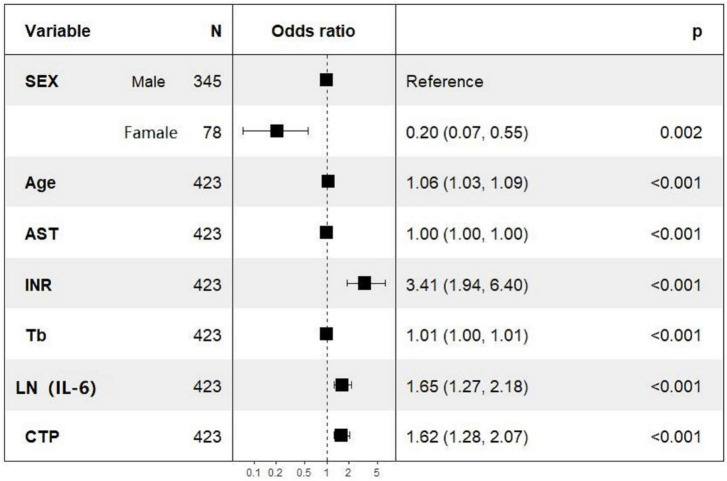
The forest map represents the result of multivariate logistic regression.

Univariate logistic regression analysis of 28-day mortality showed that gender, age, AST, TBIL, INR, PT, LN (IL-6), Child–Pugh score, MELD score, and MELD-Na score were related risk factors (*P* < 0.05) (Table 3). The parameters with *P* values less than 0.05 in the univariate logistic regression were included in the multivariate logistic regression analysis, and the results showed that gender, age, AST, TBIL, INR, CTP, and LN (IL-6) were independent risk factors for 28-day mortality. In particular, the OR of LN (IL-6) was 1.660 with a 95% CI of 1.260–2.190. The multivariate logistic regression results are shown in the forest plot in Figure 2.

**TABLE 3:** Univariate and multivariate analyses.

Variable	Univariate		Multivariate	
		
	OR (95% CI)	*P*-value	OR (95% CI)	*P*-value
Sex (male)	7.21 (2.01–25.83)	**0.002**	4.800 (1.657–13.903)	**0.004**
	Reference		Reference	
Age	1.07 (1.03–1.11)	**< 0.001**	1.0361 (1.030–1.093)	**< 0.001**
Cause of liver cirrhosis	Reference			
Viral	1.58 (0.25–10.21)	0.630		
Alcoholic	2.48 (0.12–51.75)	0.557		
Combined alcoholic + viral	0.75 (0.08–6.85)	0.801		
Other	0.83 (0.05–12.82)	0.897		
Cryptogenic	1.260 (0.501–3.165)	0.623		
Cause of hospitalization	Reference			
Ascites	1.75 (0.62–4.93)	0.292		
Gastrointestinal hemorrhage	0.11 (0.01–1.37)	0.086		
Hepatic encephalopathy	0.59 (0.09–4.03)	0.588		
Infection	2.1 (0.28–15.85)	0.472		
ALT	1.00 (0.999–1.000)	0.984		
AST	1.001 (1.0002–1.002)	**0.016**	1.001 (1.001–1.002)	**< 0.001**
Albumin	1.06 (0.95–1.19)	0.289		
ALP	1.00 (0.99–1.00)	0.368		
Bilirubin	1.01 (1.001–1.01)	**0.008**	1.005 (1.002–1.008)	**< 0.001**
Creatinine	1.00 (0.99–1.01)	0.842		
INR	34.68 (2.83–425.28)	**0.006**	9.899 (1.285–76.235)	**0.028**
PT	0.80 (0.64–1.00)	**0.049**	0.899 (0.745–1.085)	0.267
Platelets	1.00 (1.00–1.01)	0.497		
WBC	1.09 (0.96–1.25)	0.194		
Na	1.05 (0.89–1.22)	0.579		
LN (IL-6)	1.75 (1.29–2.37)	**< 0.001**	1.701 (1.283–2.087)	**< 0.001**
Child–Pugh score	1.89 (1.34–2.66)	**< 0.001**	1.636 (1.283–2.087)	**< 0.001**
MELD score	1.221 (1.165–1.281)	**< 0.001**	1.037 (0.911–1.181)	0.580
MELD-Na score	1.154 (1.115–1.195)	**< 0.001**	0.969 (0.884–1.062)	0.500
NLR	1.02 (0.97–1.08)	0.393		

Bold values represent the statistically significant.

### Predictive value for mortality in liver failure patients

Receiver operating characteristic curve analysis evaluated the accuracy of CTP score, MELD score, MELD-Na score, NLR, and LN (IL-6) in predicting 28-day, 3-month, and 6-month mortality (Table 4). LN (IL-6) at admission predicted mortality at 28 days, 3 months, and 6 months in LF patients (AUROC: 0.718, 95% CI: 0.672–0.760; AUROC: 0.715, 95% CI: 0.670–0.758; and AUROC: 0.688, 95% CI: 0.642–0.732, respectively). The critical value of LN (IL-6) at 28 days was 1.98, the sensitivity was 83.51%, and the specificity was 47.55%. At 3 months, the critical value of LN (IL-6) was 2.72, the sensitivity was 57.89%, and the specificity was 74.76%. At 6 months, the critical value of LN (IL-6) was 2.72, the sensitivity was 53.44%, and the specificity was 74.66%. The CTP score, MELD score, MELD-Na score and NLR score also had predictive value for mortality at 28 days, 3 months, and 6 months in LF patients (all *P* < 0.001).

**TABLE 4:** The efficacy of the various systems for prediction 28-day, 3-month, and 6-month mortality.

	Prognostic score	ROC area	Asymptotic sig	Cut-off point	Sensitivity (%)	Specificity (%)	PLV	NLV
28-day mortality	Child–Pugh score	0.844	**<0.001**	9	91.75	61.35	0.806	0.877
	MELD score	0.852	**<0.001**	19	85.57	72.92	0.796	0.887
	MELD-Na score	0.832	**<0.001**	19	87.60	70.20	0.793	0.867
	NLR	0.738	**<0.001**	3.57	72.16	66.56	0.693	0.779
	LN (IL-6)	0.718	**<0.001**	1.98	83.51	47.55	0.672	0.760
	IL-6 model	0.927	**<0.001**	0.25	84.00	89.70	0.898	0.953
90-day mortality	Child–Pugh score	0.847	**<0.001**	9	89.47	63.43	0.809	0.880
	MELD score	0.837	**<0.001**	19	81.58	74.68	0.794	0.867
	MELD-Na score	0.831	**<0.001**	18	88.60	66.67	0.792	0.866
	NLR	0.753	**<0.001**	3.57	72.81	68.93	0.709	0.793
	LN (IL-6)	0.715	**<0.001**	2.72	57.89	74.76	0.670	0.758
6-month mortality	Child–Pugh score	0.832	**<0.001**	9	85.50	64.73	0.793	0.867
	MELD score	0.816	**<0.001**	20	73.28	80.07	0.775	0.852
	MELD-Na score	0.811	**<0.001**	18	83.97	67.81	0.770	0.847
	NLR	0.744	**<0.001**	3.57	69.47	69.86	0.699	0.785
	LN (IL-6)	0.688	**<0.001**	2.72	53.44	74.66	0.642	0.732

Bold values represent the statistically significant.

### Construction and calibration of the prognostic nomogram

**FIGURE 3:**
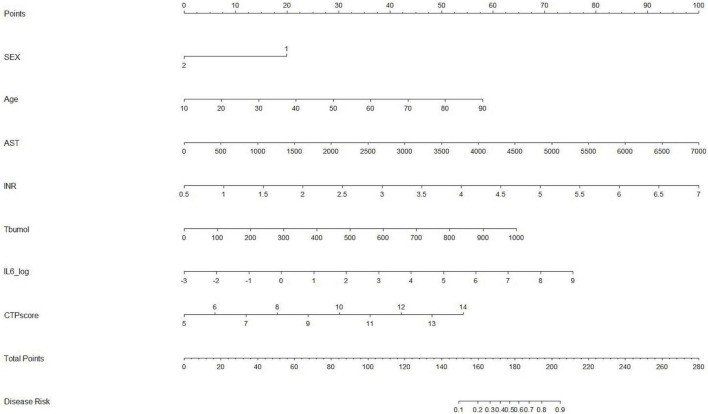
Nomogram for predicting 28-day mortality of LF patients.

**FIGURE 4:**
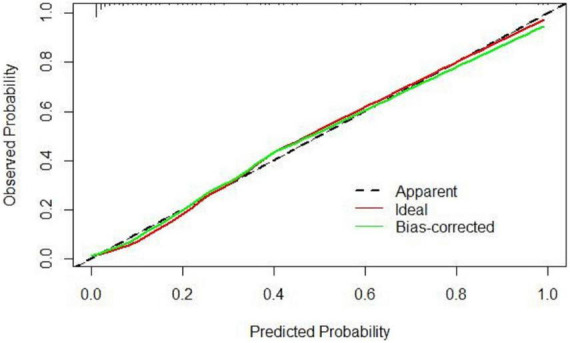
The calibration diagram verified by bootstrap re-sampling showed excellent consistency.

**FIGURE 5:**
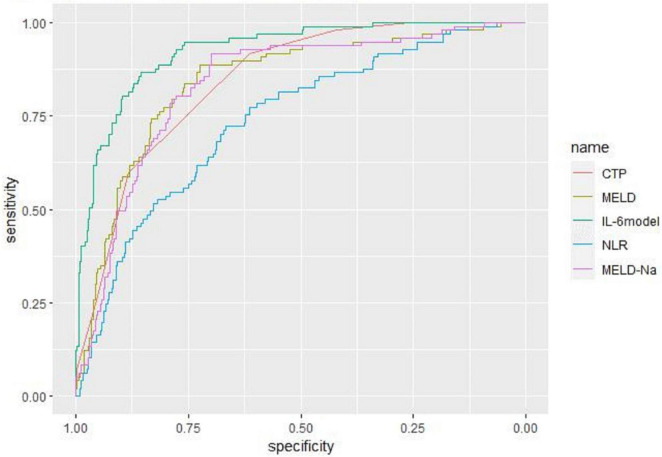
The ROC curves and comparisons of prognostic scores for 28-day.

**FIGURE 6:**
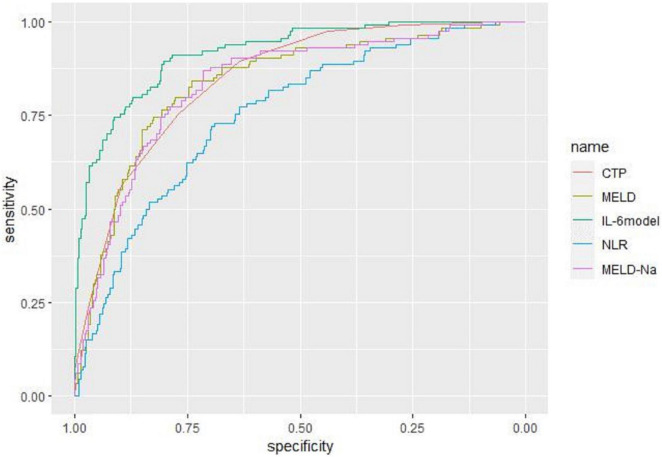
The ROC curves and comparisons of prognostic scores for 90-day.

**FIGURE 7:**
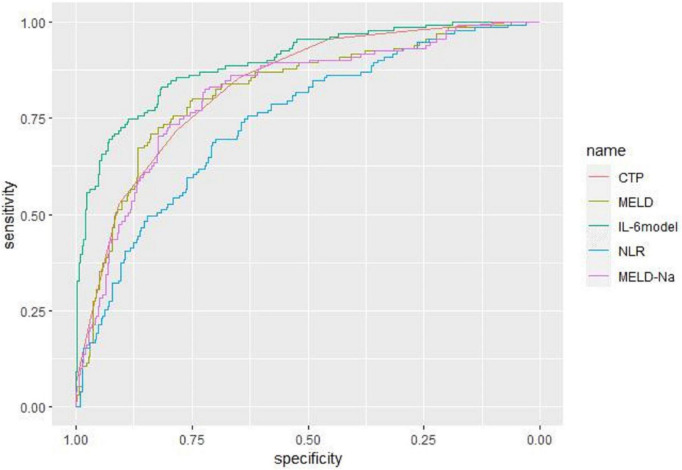
The ROC curves and comparisons of prognostic scores for 180-day.

Based on the results of multivariate logistic regression analysis, a nomogram was developed to predict 28-day mortality in LF patients (Figure 3). In particular, the nomogram was generated by assigning weighted scores to each of the seven independent prognostic parameters, including gender, age, AST, INR, TBIL, LN (IL-6), and CTP score. In the nomogram prediction, the higher the sum of the scores for each predictor is, the greater the likelihood of death. In Table 4, we note that the C-index (AUROC) of the LN (IL-6) model was 0.927 (95% CI 0.898–0.953) compared with the CTP score (0.844, 95% CI 0.806–0.877), MELD score (0.852, 95% CI 0.815–0.855) or NLR score (0.738, 95% CI 0.693–0.779), and it can be seen that the new model including IL-6 is significantly different from the traditional prognostic score (Table 5). The Hosmer–Lemeshow test showed χ^2^ to be 7.609 (*P* = 0.473). The calibration diagram verified by bootstrap resampling showed excellent consistency (Figure 4). The ROC curves and comparisons of prognostic scores are shown in Figure 5 (28-day), Figure 6 (3 months), and Figure 7 (6 months).

**TABLE 5:** IL-6 model versus other models for prediction 28-day mortality.

Pairwise comparison of ROC curves	Difference between areas (95% CI)	*Z* statistic	*P*-value
Child–Pugh vs. IL-6 model	(0.050–0.116)	4.932	**<0.001**
MELD vs. IL-6 model	(0.047–0.121)	4.463	**<0.001**
MELD-Na vs. IL-6 model	(0.054–0.128)	4.816	**<0.001**
NLR vs. IL-6 model	(0.134–0.246)	6.649	**<0.001**

Bold values represent the statistically significant.

## Discussion

Our study found that the baseline IL-6 level of LF patients who died within 4 weeks was significantly higher than that of LF patients who survived, and IL-6 is an independent factor affecting the short-term prognosis of LF. In this study, we constructed for the first time a nomogram containing IL-6 to predict 28-day mortality in patients with LF. The proposed nomogram is derived from routine laboratory data to maximize its clinical applicability and generality. The higher the IL-6 level is, the greater the likelihood of short-term death.

In a study of HBV associated with ACLF in an Asian population, the 28-day and 90-day mortality rates for rapidly progressive LF (ACLF) based on chronic liver disease were 52.1 and 69.7%, respectively (11). Here, we found a 90-day mortality rate of 27% for all LF patients, which may be due to the inclusion of patients with ALF without underlying liver disease in our study population.

Child–Turcotte–Pugh and MELD scores are the main criteria for liver disease prognosis. However, CTP covers a wide range of disease severity and subjective components, such as ascites and hepatic encephalopathy severity assessment. The MELD score is commonly used to evaluate the timing of liver transplantation in patients with end-stage chronic liver disease. In addition, in 1989, the KCH criteria was put forward for fulminant hepatic failure caused by acetaminophen (although the term has now been widely abandoned) (16) considering the causes and influence on prognosis, and it was the main LF prediction method. However, there was less research data in evaluating the Asian population with viral and (or) alcoholic etiology of ALF. Therefore, an assessment model with high prognostic accuracy is needed to assess the severity and prognosis of ALF in patients previously free of liver disease.

A total of 82.3% of LF patients in our study had viral hepatitis. Several studies have shown that HBV-ACLF can be triggered by damage caused by an overactive immune system. In patients with chronic hepatitis B, the degree of acute exacerbation of liver injury is related to cytokine levels and immune cell counts (17, 18).

In recent years, there have been many studies on the correlation between the level of inflammatory factors and the diagnosis and prognosis of LF. IL-6 family cytokines play a key role in chronic liver inflammation, a process that leads to the development of liver fibrosis, cirrhosis, and ultimately hepatocellular carcinoma (HCC). IL-6 is thought to be a key modulator linking inflammation with human fibrosis and cancer. IL-6 is the most typical cytokine in the IL-6 family involved in the development of HCC, both in experimental models and in humans. In patients with advanced HCC, elevated serum IL-6 levels are associated with poor prognosis, survival, and recurrence (19).

Therefore, some studies have explored the role of IL-6 inhibition in the prevention or treatment of inflammatory diseases. After ALF, IL-1α, IL-1β, and IL-18 were found to upregulate the proinflammatory process by significantly decreasing hepatic kappa B inhibitor levels and NF-κB pathway activation, leading to IL-6 and TNF-α secretion, which promoted apoptosis and ultimately liver injury and death in animals (20). It also inhibits the production of the inflammatory cytokines IL-1β, IL-6 (21) and TNF-α and plays a protective role in ALF.

Acute liver injury caused by virus or alcohol activates hepatic macrophages through DAMP, leading to the release of inflammatory factors. Meanwhile, hepatic macrophages produce proinflammatory factors such as TNF, IL-1β and IL-6 to activate hepatic stellate cells and destroy hepatocytes, leading to hepatic microcirculation dysfunction and increased portal vein pressure. Sustained hepatocyte injury is followed by the release of pro-inflammatory inflammatory factors (IL-1β, IL-6, IL-8, TNF-α, IFN-γ, IFN-α, IL-17) and anti-inflammatory inflammatory factors (IL-10), resulting in persistent injury, immune paralysis, systemic inflammatory response syndrome and sepsis, etc., eventually leading to organ failure (17).

However, the results are heterogeneous due to different recruitment groups and different measurement methods. In a study conducted by Wiese et al. (22), a tendency for IL-6 levels to increase with the aggravation of CTP was detected, but the difference did not reach statistical significance. Dirchwolf et al. (23) showed that IL-6 was continuously elevated during the progression of decompensated cirrhosis to ACLF, which is consistent with our original intention to investigate whether IL-6 is associated with increased mortality from LF. In addition, a linear regression model based on the CLIF-C organ failure score identified two cytokines, IL-8 and IL-6, as the markers most associated with the severity of chronic ALF (4).

Several unique characteristics of this study are as follows: (1) the etiology and main symptoms of LF are different, and some patients may progress or improve in a dynamic manner. The prognostic model of LF is universal and is not affected by miscellaneous narrow spectrum scores. (2) The probability of the occurrence of target clinical events can be generated by presenting complex mathematical formulas with simple graphs, and it can help clinicians communicate with doctors and patients more conveniently. We integrated seven parameters (including gender, a categorical variable) into the nomogram, covering some clinical characteristics of the patients more broadly, and these parameters were tested without multicollinearity. This nomogram highlights the prognostic role of IL-6 in LF, and it can better predict the 28-day mortality of LF patients at discharge. However, the included CTP score made the model lack indicators related to renal function assessment, which may be due to the low number of acute kidney injuries in our patient population. (3) Our results were basically consistent with the results of other studies. Serum IL-6 levels were correlated with the prognosis of patients with LF. These studies (7–10, 24) have identified serum IL-6 levels as a prognostic indicator of the severity of immune disorders, indicating its potential for early clinical intervention in patients with LF. To our knowledge, this study is the first to incorporate IL-6 into a prognostic model of LF. (4) A total of 45.4% of LF patients received artificial liver support therapy, such as plasmapheresis, after admission, but our study collected the serum inflammatory indicators of patients within 24 h after admission, which could exclude the influence of artificial liver support treatment on the level of inflammatory factors.

In conclusion, we made several important observations in the current study. First, serum IL-6 was significantly associated with the short-term mortality of LF. The higher the serum IL-6 level is, the greater the probability of death. Second, we established a nomogram to help clinicians estimate individual risk based on objective parameters. However, the current study is still a single-center evaluation, and it is necessary to conduct further multicenter study validation.

## Data availability statement

The raw data supporting the conclusions of this article will be made available by the authors, without undue reservation.

## Ethics statement

Ethical review and approval was not required for the study of human participants in accordance with the local legislation and institutional requirements. Written informed consent from the patients/participants or legal guardian/next of kin was not required to participate in this study in accordance with the national legislation and the institutional requirements.

## Author contributions

NX designed the study and wrote the original draft. LL analyzed the data and wrote the manuscript. YN and YZ drew figures, made tables, and revised the manuscript. NX and YZ collected the data. XZ critically revised the manuscript. All authors contributed to the article and approved the submitted version.
