# Supplementary material for: A practical nomogram based on serum interleukin-6 for the prognosis of liver failure
Source: Front Med (Lausanne). 2022 Nov 16;9:1035699. doi: 10.3389/fmed.2022.1035699 (PMC9709310; doi:10.3389/fmed.2022.1035699)
Supplement: Supplementary file 1 [file Table_1.DOCX]

**Supplement Table1 Comparing the Liver replacement therapy Between Nonsurviving Groups and Surviving Groups**

| Parameter | 28-day | | | 90-day | | | 6-month | | |
| --- | --- | --- | --- | --- | --- | --- | --- | --- | --- |
|  | Survivors | Non-survivors | P-Value | Survivors | Non-survivors | P-Value | Survivors | Non-survivors | P-Value |
| Liver replacement therapy | 122(61.6) | 76(38.4) | ＜0.001 | 113(57.1) | 85(42.9) | ＜0.001 | 102(51.5) | 96(48.5) | ＜0.001 |
